# Weighted Gene Co-Expression Network Analysis Identifies Key Modules and Hub Genes Associated with Mycobacterial Infection of Human Macrophages

**DOI:** 10.3390/antibiotics10020097

**Published:** 2021-01-20

**Authors:** Lu Lu, RanLei Wei, Sanjib Bhakta, Simon J. Waddell, Ester Boix

**Affiliations:** 1 College of Animal Science and Technology, Sichuan Agricultural University, Chengdu 610000, China; 2 Department of Biochemistry and Molecular Biology, Faculty of Biosciences, Universitat Autonoma de Barcelona, 08290 Cerdanyola del Vallès, Spain; 3 Laboratory of Omics Technology and Bioinformatics, West China Hospital, Sichuan University, Chengdu 610017, China; weijinrl@163.com; 4 Mycobacteria Research Laboratory, Department of Biological Sciences, Institute of Structural and Molecular Biology, Birkbeck, University of London, London WC1E 7HX, UK; s.bhakta@bbk.ac.uk; 5 Global Health and Infection, Brighton and Sussex Medical School, University of Sussex, Brighton BN1 9PX, UK; S.Waddell@bsms.ac.uk

**Keywords:** mycobacterium, macrophage, WGCNA, transcriptome, network analysis

## Abstract

Tuberculosis (TB) is still a leading cause of death worldwide. Treatments remain unsatisfactory due to an incomplete understanding of the underlying host–pathogen interactions during infection. In the present study, weighted gene co-expression network analysis (WGCNA) was conducted to identify key macrophage modules and hub genes associated with mycobacterial infection. WGCNA was performed combining our own transcriptomic results using *Mycobacterium aurum*-infected human monocytic macrophages (THP1) with publicly accessible datasets obtained from three types of macrophages infected with seven different mycobacterial strains in various one-to-one combinations. A hierarchical clustering tree of 11,533 genes was built from 198 samples, and 47 distinct modules were revealed. We identified a module, consisting of 226 genes, which represented the common response of host macrophages to different mycobacterial infections that showed significant enrichment in innate immune stimulation, bacterial pattern recognition, and leukocyte chemotaxis. Moreover, by network analysis applied to the 74 genes with the best correlation with mycobacteria infection, we identified the top 10 hub-connecting genes: *NAMPT*, *IRAK2*, *SOCS3*, *PTGS2*, *CCL20*, *IL1B*, *ZC3H12A*, *ABTB2*, *GFPT2,* and *ELOVL7*. Interestingly, apart from the well-known Toll-like receptor and inflammation-associated genes, other genes may serve as novel TB diagnosis markers and potential therapeutic targets.

## 1. Introduction

Tuberculosis (TB) is still a global threat and one of the leading infectious diseases according to the World Health Organisation, causing 1.5 million deaths and 10 million new cases in 2018 [1]. TB infection begins when the causal bacilli, *Mycobacterium tuberculosis* (*Mtb*), reach the alveolar air sacs of the lungs, where they invade and replicate within macrophages [2,3]. Granulomas are formed when macrophages, T lymphocytes, B lymphocytes, and fibroblasts aggregate, surrounding the infected macrophages. The granuloma prevents dissemination of the mycobacteria but provides a local environment for the infection of immune cells [3]. The formation of granulomas may also suppress host immune responses, as macrophages and dendritic cells in the granulomas are unable to present antigen to lymphocytes [4]. In addition to active disease, *Mtb* may also cause latent infection [5]. Epidemiology studies indicate that 90% of those infected with TB are asymptomatic, with only 10% lifetime risk to develop active disease [6]. Unfortunately, the risk of developing active TB increases dramatically in HIV patients [7]. The emergence of a growing number of multi-drug resistant and extensively drug-resistant TB cases makes the global situation much worse and emphasises the need for the development of new anti-TB agents able to eradicate tuberculosis [8,9]. It is also important to note that different mycobacteria induce distinct host responses, and this may contribute to the highly variable outcomes of infection ranging from asymptomatic to severe extra-pulmonary disease [10]. The impact of *Mtb* strain variation on human disease is not well established, and *Mtb* genomic diversity remains to be unambiguously correlated to the intracellular/lung lesion phenotypic diversity [11]. Understanding this host–pathogen relationship is fundamental to the development of new transformative therapies to prevent TB.

Transcriptomic profiling, the analysis of genome-wide RNA expression, is a common and powerful approach to investigate host and pathogen processes in infection models. Transcriptomic data of macrophages infected with various mycobacteria, including *M. tuberculosis H37Rv* (*Mtb-H37Rv*) [12,13], *M. tuberculosis GC1237* (*Mtb-GC1237*) [13], *M. smegmatis* [13], *M. bovis BCG* (*BCG*) [13,14,15], and *M. abscessus* (*MAB*) [16] exist in public databases; however, the comparative response behaviours of these human macrophages to these mycobacterial species has not been addressed. Moreover, *M. smegmatis*, *BCG*, *M. abscessus* as well as *M. aurum,* are frequently used as surrogate experimental models for *Mtb*, as *Mtb-H37Rv* is a slow-growing and pathogenetic species. In particular, *M. aurum* has been shown to be a good surrogate model that might be used in high-throughput screening for tuberculosis drug discovery [17]. However, we do not know to what extent the host macrophage response to *M. aurum* mimics the host response to *H37Rv* infection. Weighted gene co-expression network (WGCNA) analysis is a systems biology method for describing correlation patterns among genes in genome-wide RNA profiling experiments, from microarray samples or RNAseq samples, which has been successfully used to identify highly connected hub genes and significant modules [18]. WGCNA alleviates the multiple testing problem inherent in microarray data analysis. Instead of relating thousands of genes to a microarray sample trait, it focusses on the relationship between a few (typically less than 10) modules and the sample trait. It also enables datasets from different platforms to be compared together by focussing on groups of genes rather than on the expression of individual genes [18]. Moreover, cut-off criteria are not necessary for WGCNA, and hence, important information omitted by other statistical testing methods can be retrieved. To address the above-mentioned questions, we first captured the expression profiles of macrophages infected with *M. aurum* and then employed an integrative network-based approach to identify host response co-expression networks in other mycobacterial infection models: *Mtb-H37Rv*, *BCG*, *MAB-Rough*, *MAB-Smooth*, *M. smegmatis*, *Mtb-GC1237*, and heat-inactivated *Mtb-H37Rv*. The integration of differential gene expression, data-driven correlation, and co-expression networks enabled the reconstruction of novel signalling maps underlying the host response to mycobacterial infection. To avoid analytical bias from the individual experiments, we selected as a starting analysis material the raw mRNA sequencing data available at NCBI Sequence Read Archive or at EMBL-EBI European Nucleotide Archive.

By applying the WGCNA methodology to 198 transcriptome profiles, we identified a defined set of core genes consisting of 226 genes that were commonly regulated during host macrophage cell infection with the majority of mycobacterial species. These genes are involved in immune response stimulation by mycobacteria infection in human macrophages, which are mainly linked to Toll-like receptor and TNF signalling pathways. Of them, 74 genes were identified as hub genes representing the transcriptomic response of macrophages to mycobacterial infection. Moreover, we identified a set of 10 key hub genes that may serve to outline a common infection profile.

## 2. Results

### 2.1. Construction of Weighted Co-Expression Network

To learn about the immune response to infection by different mycobacteria, we investigated the in vitro gene regulatory response of macrophages to infection using multiple *Mtb* strains and related mycobacterial species (Table 1).

Specifically, we collected RNAseq data of human macrophage infected and/or stimulated with the following mycobacteria: *Mtb H37Rv* (pathogenic and slow growing), the common strain often used in laboratory experiments; *Mtb GC1237*, which is a clinical isolate of the virulent Beijing family; *Mycobacterium smegmatis*, which is fast-growing and non-pathogenic; *M. bovis bacillus Calmette-Guerin* (*BCG*), which is slow growing and an attenuated *Mycobacterium bovis* strain used for vaccinations; *MAB-R* and *MAB-S*, which are the rough and smooth morphotypes of the fast-growing *M. abscessus*; and heat-killed *Mtb H37Rv*. These previously published transcriptome studies were combined with our own data using *M. aurum* (PRJNA575195). The detailed features of mycobacteria are listed in Table 2.

**Figure 1:**
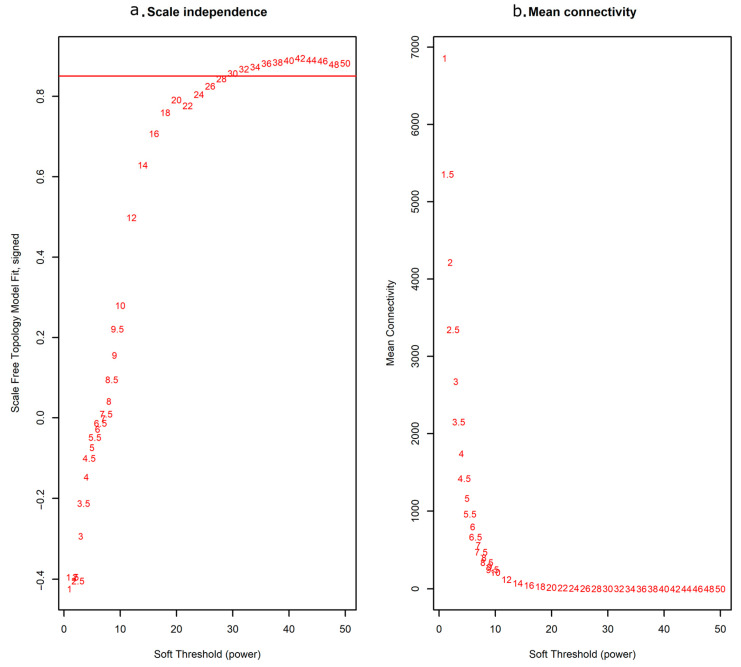
Determination of soft-thresholding power for the weighted gene co-expression network analysis (WGCNA) analysis. (**a**) Analysis of the scale-free fit index for various soft-thresholding powers (β); (**b**) Analysis of the mean connectivity for various soft-thresholding powers. In all, 30 was the most fit power value.

**Figure 2:**
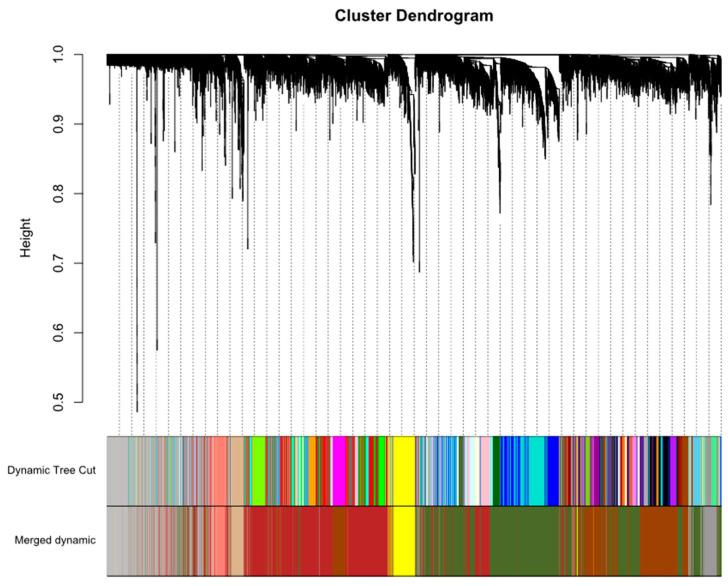
Construction of co-expression modules by WGCNA of macrophage transcriptional responses to mycobacterial infection. The cluster dendrogram of genes. Each branch in the figure represents one gene, and every colour below represents one co-expression module.

To construct the gene co-expression networks, all data were downloaded from the Gene Expression Omnibus (GEO) database, including our own unpublished experimental data, as detailed in Table 1 and Table S1. Raw data were preprocessed identically using R for background correction and normalisation. A total of 11,533 genes (Appendix A (Additional File 1)) for 198 samples were used to build a WGCNA. First, genes and conditions were clustered using the flashClust function (see Figure S1, where information on infection status, infection time, or infection type/different bacterial strains is indicated). Selection of the soft-thresholding power is an important step when constructing a WGCNA [22]. We performed the analysis of network topology for thresholding powers from 1 to 50 and identified the relatively balanced scale independence and mean connectivity of the WGCNA. As shown in Figure 1, power value 30, which was the lowest power for the scale-free topology fit index on 0.85, was selected to produce a hierarchical clustering tree (dendrogram) of 11,533 genes. From the WGCNA analysis, we identified 47 distinct modules with mergeCutHeight of 0.3 (Figure 2).

### 2.2. Correlation between Modules and Identification of Key Modules

**Figure 3:**
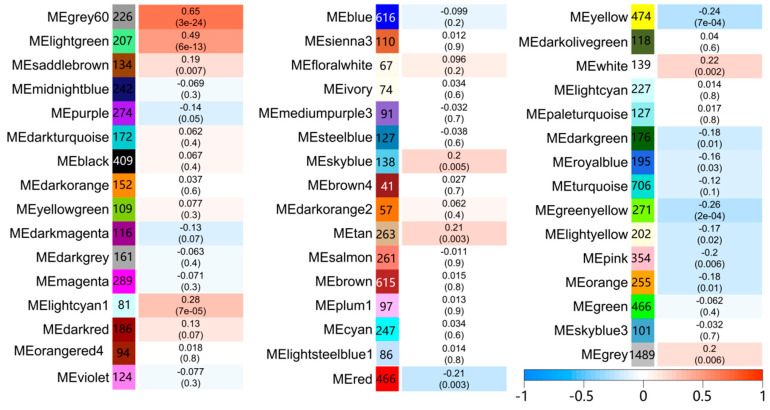
Heatmap of the correlation between module eigengenes and mycobacterial infection status. The colour of each cell at the row–column intersection indicates the correlation coefficient between the gene module and mycobacterial infection. The number of genes in each module is indicated on the left. The value of the correlation coefficient and significance between each module and infection status are indicated.

**Figure 4:**
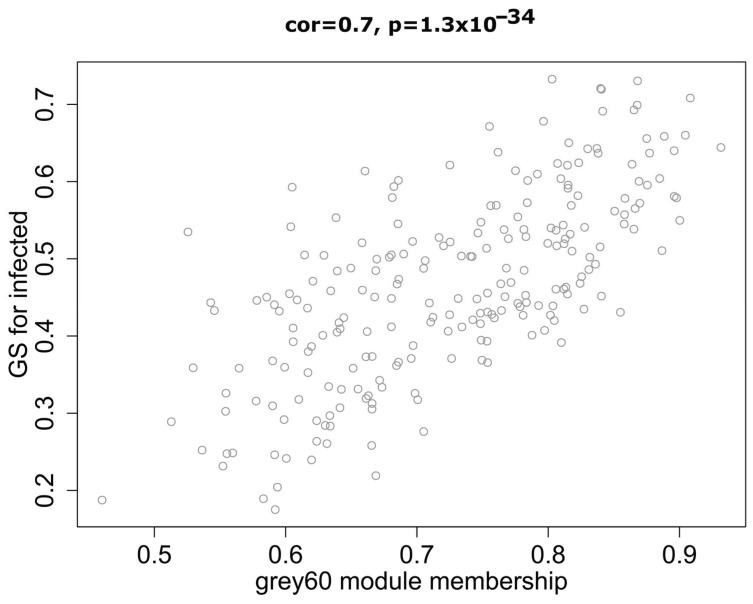
Scatter plot of module eigengenes in the grey60 module that was significantly associated with macrophage response to mycobacterial infection.

In WGCNA, modules are defined as clusters of highly interconnected genes; genes within the same cluster have high correlation coefficients. The module eigengene (ME) is representative of the corresponding module’s gene expression profile correlated with a defined trait (infection status). We analysed the interaction relationships of the 47 modules and plotted the network heatmap (Figure S2). The results showed that each module was an independent validation from the others, which indicated a high level of independence among the modules and relative independence of gene expression within each module. Moreover, we calculated eigengenes and clustered them according to their correlations. The results demonstrated that the 47 modules were mainly divided into two clusters (Figure S3). As shown in Figure 3, we found that several modules were significantly correlated with the infection status (significance value < 0.001), which was defined as presence or absence of mycobacterial infection. Among them, the grey60 module showed the highest correlation (r value) compared with the other modules. Therefore, grey60 was considered as the most relevant differentially expressed gene module across these diverse mycobacteria and variety of host macrophage cells exposed to infection. Figure 4 illustrates the correlation between module membership and gene significance in the grey60 module. The results indicated that genes in the grey60 module likely play an important role in responding to mycobacterial infection in macrophages.

### 2.3. Functional Enrichment and Identification of Hub Genes

**Figure 5:**
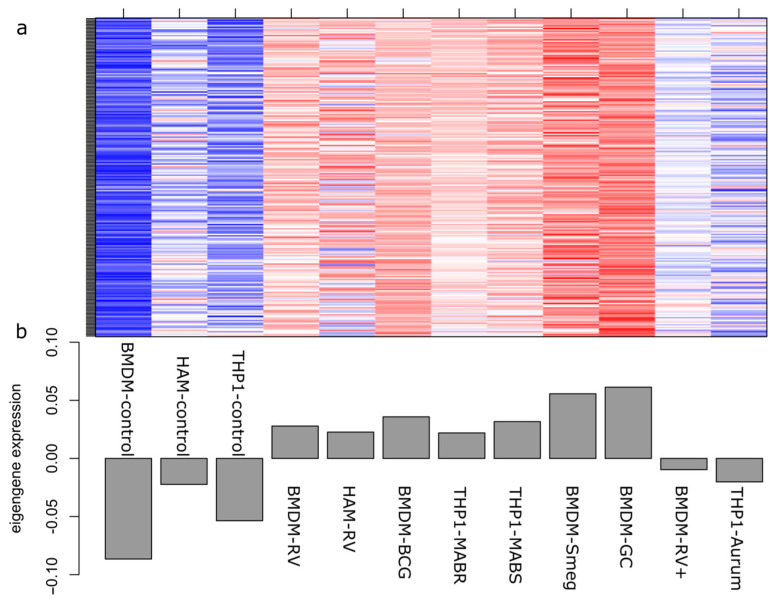
Heatmap and eigengene expression profile for grey60 module, induced by macrophages after mycobacterial infection. (**a**) The heat map shows the relative expression of each of the 226 genes; (**b**) Plot of eigengene values for the grey60 module. The y-axis indicates the value of the module eigengene; the x-axis indicates sample type. RV, *Mtb-H37Rv*; BCG, *M. bovis-BCG*; MABR, *M. abscessus-rough*; MABS, *M.abscessus-smooth*; Smeg, *M. smegmatis*; GC, *Mtb-GC1237*; RV+, heat inactivated *Mtb-H37Rv*; Aurum, *M. aurum*; Control, uninfected control macrophages.

Following this observation, we inspected in detail the composition of the grey60 module. The module is correlated with mycobacterial infection status and contains 226 genes, the expression of each gene is listed in Appendix B (Additional File 2). Appling Kyoto Encyclopedia of Genes and Genome (KEGG) and Gene Ontology (GO) enrichment analysis, we found these 226 genes significantly enriched into pathways such as Cytokine-cytokine receptor interaction, mitogen-activated protein kinase (MAPK) signalling, toumor necrosis factor (TNF) signalling, phosphatidylinositol 3-kinase (PI3K)-protein kinase B (Akt) signalling, interleukin 17 (IL-17) signalling, and biological processes such as response to lipopolysaccharides, response to molecule of bacterial origin, and positive regulation of peptidyl–tyrosine phosphorylation, respectively (full list included in Appendix C (Additional File 3)). A heatmap of the weighted relative expression of each gene in the grey60 module (Figure 5) revealed that many module genes were strongly expressed after mycobacteria infection compared with the uninfected control groups. Among the three macrophage controls, blood monocyte-derived macrophage (BMDM) had the lowest expression of these genes, followed by the THP1 and human alveolar macrophage (HAM) cells. Among the different mycobacterial infections, *Mtb GC1237* and *M. smegmatis* caused the highest upregulation of these genes and were followed by *BCG*, *Mtb H37Rv*, *MABS*, *MABR*, heat inactivated *Mtb H37Rv*, and *M. aurum*.

**Figure 6:**
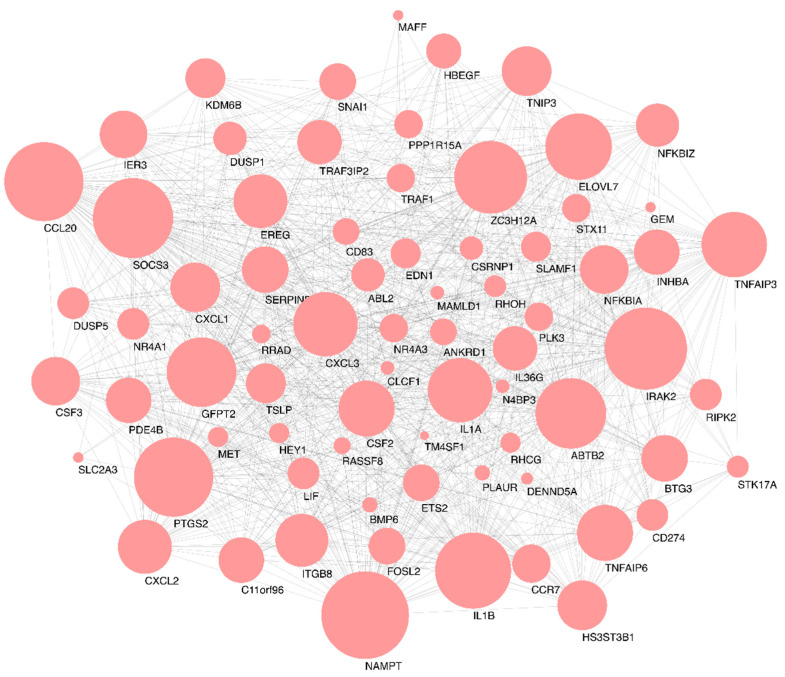
Identification of hub genes in the grey60 module induced by macrophages after mycobacterial infection. The interaction network of the selected 74 genes from the 226-gene grey60 module built by *Network Analyst*. Size of the circle is relative to number of the connections.

**Figure 7:**
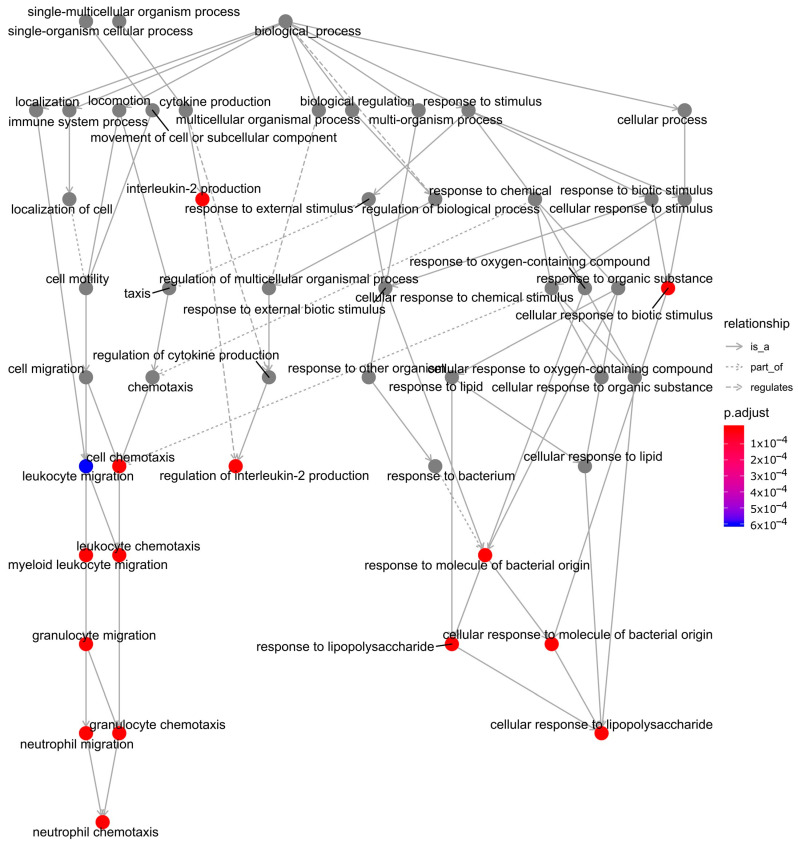
Biological process (GO) enrichment analysis of 74 genes in the grey60 module. The enriched biological processes were coloured in red or blue (according to adjusted p-values) in comparison to the connected GO terms, which are shown in grey.

Out of the 226 genes from the grey60 module, 74 genes showed the best linear correlation with mycobacteria infection (Figure 4, see the full gene list in Appendix D (Additional File 4)). Thus, those 74 genes were used as candidates for hub gene identification analysis by applying the *NetworkAnalyst* platform to map the protein-protein interactions and visualised them as networks in Cytoscape (Figure 6). The first 10 genes in the grey60 module according to their interaction degree, connected to most of the other genes within the module, were highlighted as main hub genes: nicotinamide phosphoribosyltransferase *(NAMPT)*, interleukin 1 receptor associated kinase 2 (*IRAK2)*, suppressor of cytokine signalling 3 (*SOCS3)*, prostaglandin-endoperoxide synthase 2 (*PTGS2)*, C-C motif chemokine ligand 20 (*CCL20)*, interleukin 1 beta (*IL1B)*, zinc finger CCCH-type containing 12A *(ZC3H12A)*, ankyrin repeat and BTB domain containing 2 (*ABTB2)*, glutamine fructose 6 phosphate transaminase 2 (*GFPT2),* and elongation of very long chain fatty acids protein 7 (*ELOVL7)*. We also performed enrichment analysis to explore the biological processes (BP) and pathways in which the key module was involved using the 74 genes that showed better linear correlation within the grey60 module. GO enrichment of BP was conducted by R programme clusterProfiler, as detailed in Figure 7. The findings are consistent with the results using all 226 genes in the grey60 module (Appendix C (Additional File 3)). The results highlight that genes in the grey60 module were enriched in innate immune stimulation responses, such as pathogen-associated molecular patterns (PAMPs), cytokine secretion, and immune cell migration, all of which were positively correlated with mycobacterial infection.

## 3. Discussion

The interaction between macrophage and bacterium is central to the immunopathology of mycobacterial diseases; a deeper understanding of this interplay will identify new treatment strategies. Here, we aimed to address the question of whether different mycobacteria similarly modulate the host response of macrophages. Direct comparison of the differentially expressed genes (DEGs) of macrophages infected with different mycobacteria identified from different individual studies was not appropriate to meet this goal, as the analyses were performed differently by each group and, more fundamentally, DEG analysis neglects any key but minor changes in gene expression. For example, when we first explored the transcriptional change of THP1-derived macrophages challenged with *M. aurum* by DEG analysis, we only identified 27 differentially expressed genes in comparison with uninfected control macrophages (Appendix E (Additional File 5)). The most up-regulated and down-regulated genes were *TMEM189-UBE2V1* and *RPS18*, respectively. *TMEM189-UBE2V1* is related to the IL-1 signalling pathway [23,24]; however, standard DEG analysis did not identify any further IL-1 related genes. To note, *M. aurum* shares a high similarity with *Mtb* at the genomic level [25], and most of the genes reported to be associated with drug resistance are common [26].

To compare between published macrophage transcriptome signatures and to overcome the DEG analysis drawback of neglecting minor changes in gene expression, we applied WGCNA to identify a core set of macrophage mycobacterial-responsive genes, contrasting the RNA profiles of macrophages responding to different mycobacterial infections including seven mycobacterial exposures and three types of macrophages. These mycobacteria are known to differ in pathogenicity, growth rate, morphology, and cell wall lipid composition (Table 2). The selected mycobacterial species and macrophage types represent most of the working models that have been widely used for Mtb infection characterisation [3,15,27].

In our study, by WGCNA analysis of the 198 transcriptome samples, we can categorise 11,533 genes into 47 modules according to their expression pattern (Figure 2). Among the defined modules, we identified a module (grey60), consisting of 226 genes, which strongly correlates with mycobacterial adaptations to infection (Figure 4). The grey60 module is mostly enriched with genes from biological processes such as response to pathogen-associated molecules, cytokine secretion, and chemoattractant release (Figure 7). Specifically in the module, we find genes induced in response to infection such as *IL1B*, *TNF*, *IL6*, *IL12B*, *NFKBIA*, *JUN,* and *MAP3K8*, which are related to Toll-like receptor signalling (Appendix B (Additional File 2)). To note, *IL1B*, *TNF*, *IL1A*, *IL6*, *IL23A*, *IL12B*, *PLK3*, and *IRAK2* were already reported as *Mtb*-responsive genes [27,28,29,30,31,32]. Next, by protein interaction network analysis, we identified a set of hub genes that represent the general response of macrophage infected with different mycobacteria, the top 10, connecting the genes in the module together, were *NAMPT*, *IRAK2*, *SOCS3*, *PTGS2*, *CCL20*, *IL1B*, *ZC3H12A*, *ABTB2*, *GFPT2,* and *ELOVL7.* Of them, *IRAK2* and *IL1B* are well known to be related to Toll-like receptor signalling pathways [33]; *SOCS3*, *PTGS2*, *CCL20,* and *IL1B* are linked to TNF signalling pathways [34]; *ZC3H12A* and *PTGS2* are involved in cellular inflammatory responses [35]; and *GFPT2*, *NAMPT*, *ABTB2,* and *ELOVL7* are related to cell cycle dysregulation, tissue damage, and autophagy [36]. *IFN-γ*, *IP-10*, *CRP*, *TNF-α*, *CCL4*, *IL1β,* and *TLR4* have been associated with high accuracy to TB [37,38]. Genes involved in NAD+ biosynthesis, for example, *NAMPT*, were strongly upregulated during *Mtb* infection [39]. The current data emphasise the previously suggested key role of the IFN pathway in the macrophage response to *Mtb* and successful outcome of infection [39,40]. Interestingly, some of these genes are involved in nucleic acid or lipid turnover and are also activated during other diseases, such as autoimmunity or cancer [36,40,41,42,43,44,45]. Our results back up very recent studies of *Mtb* modulation of host cell metabolism pathways [39]. Moreover, we note here the role of cell migration factors in TB pathogenesis, besides cell response to bacteria, as the genes in grey60 split into the two directions (Figure 7). In addition, interferon-γ release assay (IGRA), based on IFN-γ response, is widely used in TB diagnosis [46]. Except for the well-known TB markers such as *IRAK2*, *SOCS3*, *PTGS2*, *CCL20*, and *IL1B*, the other hub genes we identified here, *NAMPT*, *ZC3H12A*, *ABTB2*, *GFPT2,* and *ELOVL7*, may be useful as new biomarkers for TB diagnosis and as therapeutic targets for host-directed strategies. The present results based on the host metabolism can provide a complementary approach to other studies strictly focused on the mycobacterial response to available drugs in the market [47,48,49,50,51]. Hopefully, the dual sequencing of host–pathogen models [52] will help to unravel the key genes associated to both virulence and host response to infection and set the path to a better tailored drug design.

We conclude here that different mycobacterial infection models can trigger a common core set of genes related to macrophage activation and migration, which is separate from macrophage stimulation with heat-killed *Mtb*. However, we cannot disregard the inherent differences between mycobacteria strains and macrophage type, as shown in Figure 6. Control uninfected BMDM had the lowest expression of this gene module, HAM had the highest level of expression of these genes, whereas THP1 showed a medium profile. The three studied macrophage types include two primary macrophage cells: blood monocyte-derived macrophages and alveolar macrophages, together with an immortalised monocyte-like cell line (THP1). Alveolar macrophages are natural mature macrophages resident in the lung and are among the first immune cells to encounter invading mycobacteria, while blood monocytes and THP1 cells need to be activated to become macrophages. We cannot disregard the key differences between immortalized THP1 cells and their considered physiological counterparts. For example, THP-1 cells, when compared with monocytes, are far less responsive to some pathogen recognition patterns [53]. Therefore, our results emphasise once more the importance of selecting the appropriate cell infection model to suit each study. Comparing the different mycobacteria strains, *Mtb H37Rv*, *Mtb GC1237*, *M. bovis BCG*, *M. smegmatis*, *MABS,* and *MABR* showed similar induction of genes in the grey60 module, while infection with *M. aurum* or stimulation with heat-inactivated *Mtb H37Rv* resulted in a lower magnitude of upregulation of these genes. The two species, *M. aurum* and *M. smegmatis*, have been regarded as ideal surrogates for mimicking tuberculosis in drug development, both demanding a shorter time of culture and less stringent biosafety laboratory conditions [25,54].The differential regulation of the gene module identified here by all mycobacteria highlights the potentiality of some alternative infection models to live *Mtb* infection.

## 4. Materials and Methods

### 4.1. RNA Sequencing of Human Monocyte-Derived Macrophage (THP-1) Infected with M. Aurum

*M. aurum* (NCTC 10437) infection of human monocytic macrophage cell line THP-1 (NCTC #88081201) were performed as previously described; triplicate biological replicates were performed [55]. After 24 h infection, uninfected and infected macrophage cells were washed 3 times with prewarmed PBS and collected by cell scrapper for RNA extraction. Total RNA was extracted using *mir*Vana^TM^ miRNA Isolation Kit as described by the manufacturer (Ambion, Life Technologies, AM1560). RNA purity was determined by spectrophotometry, and RNA integrity was analysed using Agilent 2100 Bioanalyzer and calculated as an RNA integrity number. Following RNA extraction, RNA sequencing libraries were prepared according to protocols provided by Illumina. 50 bp-long single-end sequencing was carried out in an Illumina HiSeq2000 sequencer with a depth of >20 million reads per sample at the CRG genomics (Centre for Genomic Regulation, Barcelona) facility. The raw transcriptomic data were uploaded in NCBI Sequence Read Archive: PRJNA575195. After sequencing, quality assessment of reads was carried out using FastQC [56] to assess the distribution of Phred quality scores and mean percentage GC content across each read. Adapters were trimmed using Trimmomatic [57]. Trimmed reads were aligned to the latest human genome assembly (version GRCh38) using Hisat2 [58]. Aligned reads were converted and sorted in the SAM file format using Samtools, providing the sorted BAM file. Stringtie was used to count the number of reads mapping to the GTF file of the annotated genome using BAM file as input [59]. Following, DEseq2 was used to reveal differently expressed genes (DEGs) using the read count as input file, and the results of all statistical tests were adjusted for multiple testing (0.05) [60].

### 4.2. Data Collection and Sample Processing for WGCNA

Mycobacterial macrophage infection gene expression studies were reviewed and filtered based on the inclusion–exclusion criteria of the following: (1) human macrophage; (2) at least 2 h post infection time points; (3) minimum sample size of 3 control/3 experimental. After filtering, 6 genome-wide expression studies were selected, including our own transcriptome of *M. aurum*-infected macrophages from this study, PRJNA575195. As shown in Table 1, these datasets involved three different macrophage cell types (BMDM, THP1, HAM) infected with 8 different mycobacteria (*Mtb-H37Rv*, *BCG*, *MAB-S*, *MAB-R*, *M. smegmatis*, *Mtb-GC1237*, *M. aurum*, and heat-inactivated *Mtb-H37Rv*). All data were generated using a whole genome platform (RNAseq). In total, 198 samples were analysed, as summarised in Table 1 and detailed in full in Table S1. Ensembl IDs from RNAseq were converted into gene symbols and merged gene expression into two groups. For sequencing data, genes with less than 10 average counts per million were filtered out of the analysis. Then, the expression data were transformed using log2 transformation to normalise the data [61]. The well-established ComBat procedures in SVA packages were performed to reduce the potential study-specific batch effect [62]. Four samples were detected as outliers and filtered out using flashClust [63] (cluster dendrogram detailed in Figure S1).

### 4.3. Construction of Weighted Gene Co-Expressed Networks

We performed WGCNA to identify the gene modules of interest from the assembled dataset using R package WGCNA [18]. The standard WGCNA procedure generates a squared adjacency matrix, between genes, based on their correlation [64]. In the present study, the absolute value of the Pearson correlation between the expression profiles of all candidate genes was determined for the 11532 most varying non-redundant transcripts, which was transformed into a connection strength measure by using a power function (connection strength (i,j) = |correlation(i,j)|^ β). The scale-free topology criterion was applied to determine the lowest soft threshold power guaranteeing a scale-free topology fit (R^2^ > 0.85) [18]. To group genes with coherent expression profiles into modules, we used the WGCNA R packages average lineage hierarchical clustering to measure the dissimilarity by topological overlapping. The co-expression modules were constructed using the automatic network construction function blockwiseModules with the following settings: power, 30; degree of similarity, 0.75; minModuleSize, 30; and TOMType, signed. All other parameters of the modules were set to default [18]. Gene significance (GS) was defined as the log10 transformation of P-value (GS = lgP) in the linear regression between gene expression and sample information. In addition, module significance (MS) was defined as the average GS for all the genes in a module. Modules with significance less than 0.0001 and an absolute value of correlation coefficient higher than 0.5 were recognised as significantly associated modules.

### 4.4. Functional Enrichment of Recurrence-Associated Modules

The significantly associated modules were applied to GO and KEGG databases for function and pathway enrichment analyses [65]. P-Value corrected by Benjamin–Hochberg FDR less than 0.05 was the threshold used to discriminate significant terms.

### 4.5. Identification of Hub Genes in Key Modules

Hub genes that are highly interconnected with module nodes were considered as functionally significant. A protein–protein interaction network was constructed using *NetworkAnalyzer* [66] for the significantly associated module. The intramodular connectivity was calculated to identify hub genes (qvalue < 0.01).

## 5. Conclusions

In this work, we compared the transcriptomic responses of different types of macrophages infected with seven different mycobacterial strains: four that are pathogenic and survive intracellularly and three that are non-pathogenic surrogates. By weighted gene co-expression network analysis (WGCNA) of 198 sample profiles, we identified a specific 226-gene module enriched with genes related to bacterial unique pattern recognition, cytokine secretion, and leukocyte chemotaxis. We also defined by protein–protein network analysis a core set of genes that may be used as novel diagnosis markers and therapeutic targets. We also observed that the magnitude of expression of this immune cell response signature differed between the mycobacteria evaluated. Results highlight the close connection between the host immune system and pathogenicity, and suggest new targets for host-directed therapies.

## Figures and Tables

**Table 1:** Human macrophage gene expression profiles by RNAseq.

Bioproject	Host Cell	Mycobacterium	MOI	POI/h	Ensemble ID	Libraries	Reference ^2^
PRJNA295153	THP1 ^1^	*MAB*	10	1 h, 4 h, 24 h	21657	27	[16]
PRJNA355844	BMDM M1	*BCG*	0.18	24 h	25343	15	[19]
	BMDM M2						
PRJNA471095	HAM	*Mtb-H37Rv*	2	2 h, 24 h, 72 h	20774	36	[20]
	BMDM						
PRJNA495462	BMDM	*Mtb-H37Rv*	0.5	18 h	39354	6	[21]
PRJNA575195	THP1	*M. aurum*	10	24 h	58233	6	
PRJNA279959	BMDM	*Mtb-H37Rv*	2	4 h, 18 h, 48 h	12728	108	[13]
		*H37Rv +*					
		*M. smegmatis*					
		*Mtb-GC1237*					
		*BCG*					

^1^ Abbreviation used: THP1, THP1 activated macrophage; BMDM, blood monocyte-derived macrophage, including type 1 (indicated as BMDM-M1) and type 2 (indicated as BMDM-M2); HAM, human alveolar macrophage. *Mtb-H37Rv*, *Mycobacterium tuberculosis H37Rv*; *MAB*, *Mycobacterium abscessus*; *BCG*, *M. bovis Bacillus Calmette–Guérin* (*BCG*); *M. aurum*, *Mycobacterium aurum*; *H37Rv+*, heat-inactivated *Mtb-H37Rv*; *M. smegmatis*, *Mycobacterium smegmatis*; *Mtb-GC1237*, *Mycobacterium tuberculosis GC1237*; ^2^ including two cell lines, MAB-smooth and MAB-rough. ^2^ See Table S1 for a list of all accession numbers corresponding to each Bioproject.

**Table 2:** Characteristics of studied mycobacteria.

Strain	Growth Rate	Pathogenicity	Morphology	Note
*Mycobacterium tuberculosis Mtb H37Rv*	Slow growth	Tuberculosis	Rough	Laboratory strain
*Mycobacterium tuberculosis GC1237*	Slow growth	Tuberculosis	Rough	
*M. bovis Bacillus Calmette–Guérin* (*BCG*)	Slow growth	Non-pathogenic	Rough	An attenuated strain of *M. bovis*, used as a vaccine for TB
*M. smegmatis*	Fast growth	Non-pathogenic	Rough	
*M. abscessus Rough* (*ABR*)	Fast growth	Pathogenic	Rough	
*M. abscessus Smooth* (*ABS*)	Fast growth	Pathogenic	Smooth	
*M. aurum*	Fast growth	Non-pathogenic	Smooth	

## Data Availability

The transcriptomic data of *M. aurum* infected macrophage presented in this study are openly available in NCBI Sequence Read Archive: PRJNA575195. The other data PRJNA295153, PRJNA355844, PRJNA471095, PRJNA495462, and PRJNA279959 were downloaded from NCBI SRA.

## Supplementary Materials

The following are available online at https://www.mdpi.com/2079-6382/10/2/97/s1, Table S1. Data information. Figure S1. Dendrogram of all samples. The leaves of the tree correspond to each sample, clustering expression ratios from 11,533 genes. The first colour band underneath the tree indicates which samples appear to be outlying (the outliers, coloured in red, were excluded from the following analysis), the second colour band represents the infection time. Similarly, the remaining bands mark the bacterial trait (different mycobacteria), cell (different macrophage type), infection status (infected or control, infected coloured in red), and each combination of macrophage cell type and mycobacterial specie. RV, *Mtb-H37Rv*; BCG, *M. bovis-BCG*; MABR, *M. abscessus-rough*; MABS, *M.abscessus-smooth*; Smeg, *M. smegmatis*; GC, *Mtb-GC1237*; RV+, heat inactivated *Mtb-H37Rv*; Aurum, *M. aurum*. Figure S2. Interaction relationship analysis of co-expressed genes. Different colours of horizontal axis and vertical axis represent different gene modules. The brightness of yellow to red of the intersects represent the degree of connectivity between different modules. There was no significant difference in interactions among different modules, indicating a high-scale independence degree among these modules. Figure S3. Cluster dendrogram of module eigengenes.

## Appendix A

Additional file 1. Input dataset for WGCNA. The file contains the batch-corrected log2 counts per million for the 11,533 Ensembl genes analysed in this study for each of the 198 samples.

## Appendix B

Additional file 2. Excel of relative expression of genes in grey60 module.

## Appendix C

Additional file 3. KEGG and GO enrichment of genes in grey60 module.

## Appendix D

Additional file 4. Gene list of 74 candidate hub genes in the grey60 module.

## Appendix E

Additional file 5. Differentially expressed genes triggered by *M. aurum* infection in THP1 macrophages.

## References

[1] WHO (2019). Global Tuberculosis Report 2019.

[2] Gupta A., Kaul A., Tsolaki A.G., Kishore U., Bhakta S. (2012). Mycobacterium tuberculosis: Immune evasion, latency and reactivation. Immunobiology.

[3] Ferluga J., Yasmin H., Al-Ahdal M.N., Bhakta S., Kishore U. (2020). Natural and trained innate immunity against Mycobacterium tuberculosis. Immunobiology.

[4] Tufariello J.M., Chan J., Flynn J.L. (2003). Latent tuberculosis: Mechanisms of host and bacillus that contribute to persistent infection. Lancet Infect. Dis..

[5] Dey B., Bishai W.R. (2014). Crosstalk between Mycobacterium tuberculosis and the host cell. Semin. Immunol..

[6] Connell D.W., Berry M., Cooke G., Kon O.M. (2011). Update on tuberculosis: TB in the early 21st century. Eur. Respir. Rev..

[7] Corbett E.L., Watt C.J., Walker N., Maher D., Williams B.G., Raviglione M.C., Dye C. (2003). The growing burden of tuberculosis: Global trends and interactions with the HIV epidemic. Arch. Intern. Med..

[8] Lohrasbi V., Talebi M., Bialvaei A.Z., Fattorini L., Drancourt M., Heidary M., Darban-Sarokhalil D. (2018). Trends in the discovery of new drugs for Mycobacterium tuberculosis therapy with a glance at resistance. Tuberculosis.

[9] Lu L., Li J., Moussaoui M., Boix E. (2018). Immune Modulation by Human Secreted RNases at the Extracellular Space. Front. Immunol..

[10] Coscolla M., Gagneux S. (2010). Does *M. tuberculosis* genomic diversity explain disease diversity?. Drug Discov. Today Dis. Mech..

[11] Correa-Macedo W., Cambri G., Schurr E. (2019). The Interplay of Human and Mycobacterium Tuberculosis Genomic Variability. Front. Genet..

[12] Jhingan G.D., Kumari S., Jamwal S.V., Kalam H., Arora D., Jain N., Kumaar L.K., Samal A., Rao K.V.S., Kumar D. (2016). Comparative Proteomic Analyses of Avirulent, Virulent, and Clinical Strains of Mycobacterium tuberculosis Identify Strain-specific Patterns. J. Biol. Chem..

[13] Blischak J.D., Tailleux L., Mitrano A., Barreiro L.B., Gilad Y. (2015). Mycobacterial infection induces a specific human innate immune response. Sci. Rep..

[14] Nalpas N.C., de Park S., Magee D.A., Taraktsoglou M., Browne J.A., Conlon K.M., Rue-Albrecht K., Killick K.E., Hokamp K., Lohan A.J. (2013). Whole-transcriptome, high-throughput RNA sequence analysis of the bovine macrophage response to Mycobacterium bovis infection in vitro. BMC Genom..

[15] Malone K.M., Rue-Albrecht K., Magee D.A., Conlon K., Schubert O.T., Nalpas N.C., Browne J.A., Smyth A., Gormley E., Aebersold R. (2018). Comparative ’omics analyses differentiate Mycobacterium tuberculosis and Mycobacterium bovis and reveal distinct macrophage responses to infection with the human and bovine tubercle bacilli. Microb. Genom..

[16] Aulicino A., Dinan A.M., Miranda-CasoLuengo A.A., Browne J.A., Rue-Albrecht K., MacHugh D.E., Loftus B.J. (2015). High-throughput transcriptomics reveals common and strain-specific responses of human macrophages to infection with Mycobacterium abscessus Smooth and Rough variants. BMC Genom..

[17] Gupta A., Bhakta S. (2012). An integrated surrogate model for screening of drugs against mycobacterium tuberculosis. J. Antimicrob. Chemother..

[18] Langfelder P., Horvath S. (2008). WGCNA: An R package for weighted correlation network analysis. BMC Bioinform..

[19] Lardone R.D., Chan A.A., Lee A.F., Foshag L.J., Faries M.B., Sieling P.A., Lee D.J. (2017). Mycobacterium bovis Bacillus Calmette–Guérin Alters melanoma microenvironment Favoring antitumor T cell responses and improving M2 Macrophage Function. Front. Immunol..

[20] Papp A.C., Azad A.K., Pietrzak M., Williams A., Handelman S.K., Igo R.P., Stein C.M., Hartmann K., Schlesinger L.S., Sadee W. (2018). AmpliSeq transcriptome analysis of human alveolar and monocyte-derived macrophages over time in response to Mycobacterium tuberculosis infection. PLoS ONE.

[21] Coya J.M., De Matteis L., Giraud-Gatineau A., Biton A., Serrano-Sevilla I., Danckaert A., Dillies M.-A., Gicquel B., De la Fuente J.M., Tailleux L. (2019). Tri-mannose grafting of chitosan nanocarriers remodels the macrophage response to bacterial infection. J. Nanobiotechnology.

[22] Zhang X., Feng H., Li Z., Li D., Liu S., Huang H., Li M. (2018). Application of weighted gene co-expression network analysis to identify key modules and hub genes in oral squamous cell carcinoma tumorigenesis. Onco. Targets. Ther..

[23] Wang F., Huang S., Gao H., Zhou Y., Lai C., Li Z., Xian W., Qian X., Li Z., Huang Y. (2020). Initial Whole Genome Sequencing and Analysis of the Host Genetic Contribution to COVID-19 Severity and Susceptibility. Cell Discov..

[24] Stelzer G., Rosen N., Plaschkes I., Zimmerman S., Twik M., Fishilevich S., Stein T.I., Nudel R., Lieder I., Mazor Y. (2016). The GeneCards Suite: From Gene Data Mining to Disease Genome Sequence Analyses. Curr. Protoc. Bioinforma..

[25] Leprae M., Phelan J., Maitra A., Mcnerney R., Nair M., Gupta A., Coll F., Pain A., Bhakta S., Clark T.G. (2015). The draft genome of Mycobacterium aurum, a potential model organism for investigating drugs against Mycobacterium tuberculosis and Mycobacterium leprae. Int. J. Mycobacteriology.

[26] Namouchi A., Cimino M., Favre-Rochex S., Charles P., Gicquel B. (2017). Phenotypic and genomic comparison of Mycobacterium aurum and surrogate model species to Mycobacterium tuberculosis: Implications for drug discovery. BMC Genom..

[27] Hickman S.P., Chan J., Salgame P. (2002). Mycobacterium tuberculosis Induces Differential Cytokine Production from Dendritic Cells and Macrophages with Divergent Effects on Naive T Cell Polarization. J. Immunol..

[28] Cooper A.M., Mayer-Barber K.D., Sher A. (2011). Role of innate cytokines in mycobacterial infection. Mucosal Immunol..

[29] Van Crevel R., Ottenhoff T.H.M., van der Meer J.W.M. (2002). Innate Immunity to Mycobacterium tuberculosis. Clin. Microbiol. Rev..

[30] Boro M., Singh V., Balaji K.N. (2016). Mycobacterium tuberculosis-triggered Hippo pathway orchestrates CXCL1/2 expression to modulate host immune responses. Sci. Rep..

[31] Dunlap M.D., Howard N., Das S., Scott N., Ahmed M., Prince O., Rangel-Moreno J., Rosa B.A., Martin J., Kaushal D. (2018). A novel role for C–C motif chemokine receptor 2 during infection with hypervirulent Mycobacterium tuberculosis. Mucosal Immunol..

[32] Volpe E., Cappelli G., Grassi M., Martino A., Serafino A., Colizzi V., Sanarico N., Mariani F. (2006). Gene expression profiling of human macrophages at late time of infection with Mycobacterium tuberculosis. Immunology.

[33] Cohen P. (2014). The TLR and IL-1 signalling network at a glance. J. Cell Sci..

[34] Xu G., Wang J., Gao G.F., Liu C.H. (2014). Insights into battles between Mycobacterium tuberculosis and macrophages. Protein Cell.

[35] Ishrat R. (2018). Stage specific classification of DEGs via statistical profiling and network analysis reveals potential biomarker associated with various stages of TB. bioRxiv.

[36] Ariel O., Gendron D., Dudemaine P.L., Gévry N., Ibeagha-Awemu E.M., Bissonnette N. (2020). Transcriptome Profiling of Bovine Macrophages Infected by Mycobacterium avium spp. paratuberculosis Depicts Foam Cell and Innate Immune Tolerance Phenotypes. Front. Immunol..

[37] Goletti D., Lindestam Arlehamn C.S., Scriba T.J., Anthony R., Cirillo D.M., Alonzi T., Denkinger C.M., Cobelens F. (2018). Can we predict tuberculosis cure? What tools are available?. Eur. Respir. J..

[38] Walzl G., Ronacher K., Hanekom W., Scriba T.J., Zumla A. (2011). Immunological biomarkers of tuberculosis. Nat. Rev. Immunol..

[39] Vrieling F., Kostidis S., Spaink H.P., Haks M.C., Mayboroda O.A., Ottenhoff T.H.M.M., Joosten S.A. (2020). Analyzing the impact of Mycobacterium tuberculosis infection on primary human macrophages by combined exploratory and targeted metabolomics. Sci. Rep..

[40] Wu K., Dong D., Fang H., Levillain F., Jin W., Mei J., Gicquel B., Du Y., Wang K., Gao Q. (2012). An Interferon-Related Signature in the Transcriptional Core Response of Human Macrophages to Mycobacterium tuberculosis Infection. PLoS ONE.

[41] Zhang W., Bouchard G., Yu A., Shafiq M., Jamali M., Shrager J.B., Ayers K., Bakr S., Gentles A.J., Diehn M. (2018). GFPT2-expressing cancer-associated fibroblasts mediate metabolic reprogramming in human lung adenocarcinoma. Cancer Res..

[42] Zhang H., Zhang N., Liu Y., Su P., Liang Y., Li Y., Wang X., Chen T., Song X., Sang Y. (2019). Epigenetic regulation of NAMPT by NAMPT-AS drives metastatic progression in triple-negative breast cancer. Cancer Res..

[43] Rienksma R.A., Suarez-Diez M., Mollenkopf H.-J.J., Dolganov G.M., Dorhoi A., Schoolnik G.K., Martins dos Santos V., Kaufmann S.H.E., Schaap P.J., Gengenbacher M. (2015). Comprehensive insights into transcriptional adaptation of intracellular mycobacteria by microbe-enriched dual RNA sequencing. BMC Genom..

[44] Mao R., Yang R., Chen X., Harhaj E.W., Wang X., Fan Y. (2017). Regnase-1, a rapid response ribonuclease regulating inflammation and stress responses. Cell. Mol. Immunol..

[45] Gleeson L.E., Sheedy F.J., Palsson-McDermott E.M., Triglia D., O’Leary S.M., O’Sullivan M.P., O’Neill L.A.J., Keane J. (2016). Cutting Edge: Mycobacterium tuberculosis Induces Aerobic Glycolysis in Human Alveolar Macrophages That Is Required for Control of Intracellular Bacillary Replication. J. Immunol..

[46] Petruccioli E., Scriba T.J., Petrone L., Hatherill M., Cirillo D.M., Joosten S.A., Ottenhoff T.H., Denkinger C.M., Goletti D. (2016). Correlates of tuberculosis risk: Predictive biomarkers for progression to active tuberculosis. Eur. Respir. J..

[47] Waddell S.J., Stabler R.A., Laing K., Kremer L., Reynolds R.C., Besra G.S. (2004). The use of microarray analysis to determine the gene expression profiles of Mycobacterium tuberculosis in response to anti-bacterial compounds. Tuberculosis.

[48] Boshoff H.I.M., Myers T.G., Copp B.R., McNeil M.R., Wilson M.A., Barry C.E. (2004). The Transcriptional Responses of Mycobacterium tuberculosis to Inhibitors of Metabolism. J. Biol. Chem..

[49] Altaf M., Miller C.H., Bellows D.S., O’Toole R. (2010). Evaluation of the Mycobacterium smegmatis and BCG models for the discovery of Mycobacterium tuberculosis inhibitors. Tuberculosis.

[50] Rampacci E., Stefanetti V., Passamonti F., Henao-Tamayo M. (2020). Preclinical Models of Nontuberculous Mycobacteria Infection for Early Drug Discovery and Vaccine Research. Pathogens.

[51] Obregón-Henao A., Arnett K.A., Henao-Tamayo M., Massoudi L., Creissen E., Andries K., Lenaerts A.J., Ordway D.J. (2015). Susceptibility of mycobacterium abscessus to antimycobacterial drugs in preclinical models. Antimicrob. Agents Chemother..

[52] Macho Rendón J., Lang B., Ramos Llorens M., Gaetano Tartaglia G., Torrent Burgas M. (2021). DualSeqDB: The host–pathogen dual RNA sequencing database for infection processes. Nucleic Acids Res..

[53] Bosshart H., Heinzelmann M. (2016). THP-1 cells as a model for human monocytes. Ann. Transl. Med..

[54] Gupta A., Bhakta S., Kundu S., Gupta M., Srivastava B.S., Srivastava R. (2009). Fast-growing, non-infectious and intracellularly surviving drug-resistant Mycobacterium aurum: A model for high-throughput antituberculosis drug screening. J. Antimicrob. Chemother..

[55] Lu L., Arranz-Trullén J., Prats-Ejarque G., Pulido D., Bhakta S., Boix E. (2019). Human Antimicrobial RNases Inhibit Intracellular Bacterial Growth and Induce Autophagy in Mycobacteria-Infected Macrophages. Front. Immunol..

[56] Babraham Bioinformatics-FastQC A Quality Control tool for High Throughput Sequence Data. http://www.bioinformatics.babraham.ac.uk/projects/fastqc/.

[57] Bolger A.M., Lohse M., Usadel B. (2014). Trimmomatic: A flexible trimmer for Illumina sequence data. Bioinformatics.

[58] Pertea M., Kim D., Pertea G.M., Leek J.T., Salzberg S.L. (2016). Transcript-level expression analysis of RNA-seq experiments with HISAT, StringTie and Ballgown. Nat. Protoc..

[59] Pertea M., Pertea G.M., Antonescu C.M., Chang T.-C., Mendell J.T., Salzberg S.L. (2015). StringTie enables improved reconstruction of a transcriptome from RNA-seq reads. Nat. Biotechnol..

[60] Love M.I., Huber W., Anders S. (2014). Moderated estimation of fold change and dispersion for RNA-seq data with DESeq2. Genome Biol..

[61] Zhai X., Xue Q., Liu Q., Guo Y., Chen Z. (2017). Colon cancer recurrence-associated genes revealed by WGCNA co-expression network analysis. Mol. Med. Rep..

[62] Zhou G., Stevenson M.M., Geary T.G., Xia J. (2016). Comprehensive Transcriptome Meta-analysis to Characterize Host Immune Responses in Helminth Infections. PLoS Negl. Trop. Dis..

[63] Horvath S. (2011). Integrated Weighted Correlation Network Analysis of Mouse Liver Gene Expression Data. Weighted Network Analysis.

[64] Botía J.A., Vandrovcova J., Forabosco P., Guelfi S., D’Sa K., Hardy J., Lewis C.M., Ryten M., Weale M.E. (2017). An additional k-means clustering step improves the biological features of WGCNA gene co-expression networks. BMC Syst. Biol..

[65] Yu G., Wang L.-G., Han Y., He Q.-Y. (2012). clusterProfiler: An R Package for Comparing Biological Themes Among Gene Clusters. Omi. A J. Integr. Biol..

[66] Su G., Morris J.H., Demchak B., Bader G.D. (2014). Biological Network Exploration with Cytoscape 3. Curr. Protoc. Bioinforma..

