# Correction: Lu et al. Weighted Gene Co-Expression Network Analysis Identifies Key Modules and Hub Genes Associated with Mycobacterial Infection of Human Macrophages. *Antibiotics* 2021, *10*, 97

**DOI:** 10.3390/antibiotics10070792

**Published:** 2021-06-29

**Authors:** Lu Lu, Ran-Lei Wei, Sanjib Bhakta, Simon J. Waddell, Ester Boix

**Affiliations:** 1 College of Animal Science and Technology, Sichuan Agricultural University, Chengdu 610000, China; 2 Department of Biochemistry and Molecular Biology, Faculty of Biosciences, Universitat Autonoma de Barcelona, 08290 Cerdanyola del Vallès, Spain; 3 Laboratory of Omics Technology and Bioinformatics, West China Hospital, Sichuan University, Chengdu 610000, China; weijinrl@163.com; 4 Mycobacteria Research Laboratory, Department of Biological Sciences, Institute of Structural and Molecular Biology, Birkbeck, University of London, London WC1E 7HX, UK; s.bhakta@bbk.ac.uk; 5 Global Health and Infection, Brighton and Sussex Medical School, University of Sussex, Brighton BN1 9PX, UK; S.Waddell@bsms.ac.uk

The authors wish to make the following corrections to this paper [1].

## Correction in Figure/Table

** Table S1 incorporates now details on library preparation and sequencing methodologies**. The updated**Table S1** appears in Supplementary File 1. The Antibiotics Editorial Office and authors apologize for any inconvenience caused by these errors in the journal editorial process and state that the scientific conclusions are unaffected. The original article has been updated.

## Text Correction

** To clarify the information one sentence in Section 2.1 was rephrased **.

A correction has been made to ***2.1 Sub-section***, ** Paragraph 3 **:

** To construct the gene co-expression networks, all data were downloaded from the Gene Expression Omnibus (GEO) database, including our own unpublished experimental data, as detailed in Table 1 and Table S1. Raw data were preprocessed identically by log2CPM transformation and normalised by Combat. A total of 11,533 genes (Appendix A (Additional File 1)) for 198 samples were used to build a WGCNA. First, genes and conditions were clustered using the flashClust function (see Figure S1, where information on infection status, infection time, or infection type/different bacterial strains is indicated). Selection of the soft-thresholding power is an important step when constructing a WGCNA [22]. We performed the analysis of network topology for thresholding powers from 1 to 50 and identified the relatively balanced scale independence and mean connectivity of the WGCNA. As shown in Figure 1, power value 30, which was the lowest power for the scale-free topology fit index on 0.85, was selected to produce a hierarchical clustering tree (dendrogram) of 11,533 genes. From the WGCNA analysis, we identified 47 distinct modules with mergeCutHeight of 0.3 (Figure 2). **

## Text Correction

** Two sentences in methodology section were modified to provide more specific information **.

A correction has been made to ***4.2 Sub-section***, ** Paragraph 1 **:

Mycobacterial macrophage infection gene expression studies were reviewed and filtered based on the inclusion–exclusion criteria of the following: (1) human macrophage; (2) at least 2 h post infection time points; (3) minimum sample size of 3 control/3 experimental. After filtering, 6 genome-wide expression studies were selected, including our own transcriptome of *M. aurum*-infected macrophages from this study, PRJNA575195. As shown in Table 1, these datasets involved three different macrophage cell types (BMDM, THP1, HAM) infected with 8 different mycobacteria (*Mtb*-H37Rv, BCG, MAB-S, MAB-R, *M. smegmatis*, *Mtb*-GC1237, *M. aurum*, and heat-inactivated *Mtb*-H37Rv). All data were generated using a whole genome platform (RNAseq). In total, 198 samples were analysed, as summarised in Table 1 and detailed in full in Table S1. Genes with less than 10 average counts per million were filtered out of the analysis. Then, the expression data were transformed using log2counts per million (CPM) transformation [61]. The well-established ComBat procedures in SVA packages were performed to reduce the batch effect from two main sources of variations, laboratory factor and infection conditions factor [62]. Four samples were detected as outliers and filtered out using flashClust [63] (cluster dendrogram detailed in Figure S1).

## Text Correction

** One sentence in methodology section was modified to provide more specific information **.

A correction has been made to ***4.3 Sub-section***, ** Paragraph 1 **:

We performed WGCNA to identify the gene modules of interest from the assembled dataset using R package WGCNA, using log2 read counts normalized for batch effects by ComBat as input (Additional file1) [18]. The standard WGCNA procedure generates a squared adjacency matrix, between genes, based on their correlation [64]. In the present study, the absolute value of the Pearson correlation between the expression profiles of all candidate genes was determined for the most varying non-redundant transcripts, which was transformed into a connection strength measure by using a power function (connection strength (i,j) = |correlation(i,j)|^ β). The scale-free topology criterion was applied to determine the lowest soft threshold power guaranteeing a scale-free topology fit (R^2^ > 0.85) [18]. To group genes with coherent expression profiles into modules, we used the WGCNA R packages average lineage hierarchical clustering to measure the dissimilarity by topological overlapping. The co-expression modules were constructed using the automatic network construction function blockwiseModules with the following settings: power, 30; degree of similarity, 0.75; minModuleSize, 30; and TOMType, signed. All other parameters of the modules were set to default [18]. Gene significance (GS) was defined as the log10 transformation of P-value (GS = lgP) in the linear regression between gene expression and sample information. In addition, module significance (MS) was defined as the average GS for all the genes in a module. Modules with significance less than 0.0001 and an absolute value of correlation coefficient higher than 0.5 were recognised as significantly associated modules.

The Antibiotics Editorial Office and authors apologize for any inconvenience caused by these errors in the journal editorial process and state that the scientific conclusions are unaffected. The original article has been updated.
